# Correlation of anti-acetylcholine receptor antibody levels and long-term outcomes of juvenile myasthenia gravis in Taiwan: a case control study

**DOI:** 10.1186/s12883-019-1397-0

**Published:** 2019-07-18

**Authors:** Cheng-Che Chou, I-Chen Su, I-Jun Chou, Jainn-Jim Lin, Shih-Yun Lan, Yi-Shan Wang, Shu-Sing Kong, Yun-Ju Chen, Meng-Ying Hsieh, Po-Cheng Hung, Huei-Shyong Wang, Min-Liang Chou, Kuang-Lin Lin, CHEESE study group

**Affiliations:** 1 Division of Paediatrics, Keelung Chang Gung Memorial Hospital, 222, Maijin Road, Keelung, Taiwan; 2 Division of Paediatric Neurology, Chang Gung Memorial Hospital and Chang Gung Children’s Hospital, Chang Gung University College of Medicine, 33305, No. 5, Fuxing St., Guishan Dist, Taoyuan, Taiwan; 3 Division of Paediatrics, Neurocritical Care Centre, Chang Gung Memorial Hospital, Taoyuan, Taiwan; 4 Division of Paediatrics, Saint Paul Hospital, 33305, No. 5, Fuxing St., Guishan Dist, Taoyuan, Taiwan; 5 Division of Paediatrics, Taipei Medical University Shuang Ho Hospital, Zhonghe Dist., New Taipei City, Taiwan

**Keywords:** Myasthenia gravis, Juvenile, Asia, Acetylcholine receptors, Graves’ disease, Outcome assessment

## Abstract

**Background:**

Myasthenia gravis is the most common disease affecting the neuromuscular junction. The most common etiology among patients with juvenile myasthenia gravis is the production of antibodies against the acetylcholine receptor. However, the clinical outcome in relation to serum levels of anti-acetylcholine receptor antibodies in juvenile myasthenia gravis has rarely been discussed. We aimed to analyze the correlation between the presence of anti-acetylcholine receptor antibodies and outcome in juvenile myasthenia gravis.

**Methods:**

Patients diagnosed with juvenile myasthenia gravis younger than of 20 years of age were retrospectively recruited from January 1995 to February 2017 in a tertiary referral medical center. According to the Myasthenia Gravis Foundation of America outcome scale, the primary outcome was complete symptom remission and cessation of medications for at least 1 year measured 2 years after diagnosis. Secondary outcome was complete symptom remission at the last outpatient clinic.

**Results:**

A total of 54 patients were followed up for over 2 years. Nine patients (9/54, 16.7%) achieved complete remission without medication use at 2 years after diagnosis. Thirteen (24.1%) patients achieved complete remission during longer follow-up periods. Those with negative anti-acetylcholine receptor antibodies were more likely to achieve complete remission at 2 years (6/15 [40%] vs. 3/39 [7.7%], 95% Confidence interval [CI] 1.670 to 38.323) and at the last outpatient clinic follow-up (8/15 [53.3%] vs. 5/39 [12.8%], 95% CI 2.367 to 20.704). Thirteen patients with comorbid autoimmune thyroid diseases were older than those without disease (11.8 ± 5.8 years old vs. 8.0 ± 6.3 years old, 95% CI 0.018 to 7.33). Moreover, patients negative for anti-acetylcholine receptor antibodies were less likely comorbid with autoimmune thyroid disease (1/35 [2.9%] vs. 12/71 [16.9%], 95% CI 0.018 to 1.161).

**Conclusions:**

Juvenile myasthenia gravis patients without anti-acetylcholine antibodies exhibited significantly increased complete remission rates and a reduced likelihood of comorbid autoimmune thyroid diseases compared with those with anti-acetylcholine receptor antibodies among Chinese.

**Electronic supplementary material:**

The online version of this article (10.1186/s12883-019-1397-0) contains supplementary material, which is available to authorized users.

## Background

Myasthenia gravis (MG) is the most common autoimmune disease affecting the neuromuscular junction. Juvenile onset myasthenia gravis (JMG) accounts for 11–24% of all patients with MG [1]. Among patients with autoimmune JMG, the most common antibody observed is the anti-acetylcholine receptor antibody. Antibody-positive patients account for 50 to 90% of JMG cases [2, 3]. Other antibodies against muscle-specific kinase (MuSK) [4], cortactin and lipoprotein receptor-related protein 4 are also reported [5]. These antibodies are used to help clinical diagnosis of myasthenia gravis; however, whether these antibodies can predict outcome is still debated.

Regarding overall outcome, JMG patients demonstrated an increased remission rate compared with adult-onset MG, ranging from 11 to 60% [1, 2, 6, 7]. Prepubertal onset MG exhibits an increased remission rate compared with postpuberty onset MG [2]. Patient weakness exclusively limited to the ocular area is associated with a better outcome compared with the presentation of general weakness [8]. The presence of antibodies other than anti-acetylcholine receptor antibodies, such as anti-MuSK antibody, is associated with poor outcome [4]. However, serum levels of anti-acetylcholine receptor antibodies in relation with the clinical outcome in JMG have rarely been discussed [1, 9]. Andrew et al. reported that remission rate did not differ between seropositive and seronegative patients [1]. However, seronegative cut-off levels were varied from 0.2 to 0.5 among the studies [1, 10, 11]. Besides, the ethnic origin of the patients influences the acetylcholine receptor antibodies serum level and clinical outcome [1, 12]. The correlation between acetylcholine receptor antibodies serum level and outcome might be different between races. Whether anti-acetylcholine receptor antibodies predict outcome in Chinese remains unknown. The aim of this study was to analyze the predictive value of the different cut-off levels of anti-acetylcholine receptor antibodies for outcome prediction among Chinese patients with JMG.

## Methods

### Setting and participants

Patients under the age of 20 years diagnosed with JMG from January 1995 to October 2015 in Chang Gung Memorial Hospital, Linko branch, which is a tertiary referral medical center, were retrospectively reviewed using medical records. The diagnosis was made by at least one of the following specialists: pediatric neurologist, ophthalmologist, or neurologist. Another pediatric neurologist would further confirm the diagnosis. JMG patients were clinically defined by the presence of at least one clinical symptoms of ptosis or proximal weakness plus positive test results for at least one of the following assessments: (1) responsive to Tensilon test or anti-cholinergic regimen, (2) decremental response to the 3 Hz repetitive stimulation test, and (3) presence of serum anti-acetylcholine receptor antibodies (≥ 0.5 nanomoles per liter [nmol/L]). Patients were excluded if electromyography or muscle biopsy reports indicated other neuromuscular diseases**.**

### Data used in the study

Data regarding race, sex, age at onset, clinical symptoms and maximum disease severity, history pharmacological treatment, family history and other interventions were collected. Age at onset was defined as the age at the time of the first manifestation of the disease or any neurological sign indicative of MG.

### Disease severity and outcome measure

Clinical severity and outcome measures were determined according to the Myasthenia Gravis Foundation of America recommendation [13]. The severities were classified as follows: Class I, ocular MG and all other muscle strength is normal; Class II, mild weakness affecting muscles other than ocular muscles; Class III, moderate weakness affecting muscles other than ocular muscles; Class IV, severe weakness affecting muscles other than ocular muscles; and Class V, intubation with or without mechanical ventilation. Primary outcome was measured based on the complete symptom remission (CSR) rate, which is the cessation of medication for at least 1 year without symptoms. The results were measured 2 years after diagnosis. The secondary outcome was the change in symptoms between initial manifestation and at the last outpatient clinic, wherein residual symptoms were observed. The change in symptoms was categorized as (1) complete remission and discontinuation of all drug therapy; (2) good improvement but continuation of drug therapy at the same or lower dosage; (3) unchanged, slight subjective and/or objective improvement but large doses of medication are necessary; or (4) worse.

### Laboratory antibody measurements

All blood specimens were analyzed in the clinical laboratory. Test results were collected from the central database. In this laboratory, internal and external quality control procedures for biochemical data yielded consistently satisfactory results. External quality control was ensured by observing the guidelines promulgated by the College of American Pathologist and the National Quality Control Program of the Government of Taiwan.

Antibodies against acetylcholine receptor antibody were tested as described previously in serum samples collected using the radioimmunoassay kit according to the RSR manufacturer’s instructions at UK (http://www.rsrltd.com). Serology tests were performed at the initial stage for diagnosis. The presence of antibodies was double checked if symptoms were not controlled during treatment course. The peak level of anti-acetylcholine receptor antibodies between diagnosis date and the end of follow-up was chosen as the primary exposure variable. Using the highest serum level, we categorized the test results as seronegative if the antibody levels were <  0.2 nmol/L, equivocal if levels were 0.2 ~ 0.49 nmol/L, and positive if levels were ≥ 0.5 nmol/L. Blood samples were examined for the presence of anti-Musk antibodies for patients with systemic myasthenia gravis with persistently negative antibodies against the acetylcholine receptor. Tests for other MG-related antibodies were not available in our center.

### Statistical analysis

Fisher’s exact test was performed to analyze overall differences in baseline characteristics among the three patient subgroups when comparing categorical variables and the correlation between anti-acetylcholine antibodies and outcome. In addition, pairwise comparisons between any two study groups were done by using Bonferroni adjustment.

To determine the optimum discriminant value of anti-acetylcholine receptor antibodies to predict the clinical outcome in JMG, we performed receiver operator characteristic (ROC) curves. Sensitivity, specificity, positive predictive values (PPV) and negative predictive values (NPV) were calculated for the outcome corresponding to different cut-off values of anti-acetylcholine receptor antibodies. Logistic regression was used for analysis of a dataset in which there are one or more independent variables that determine an outcome.

A *p*-value of less than 0.05 using two-tailed tests was considered statistically significant. Data analysis was performed using the statistical package of IBM SPSS Statistics for Windows, Version 21.0. (Armonk, NY: IBM Corp).

## Results

### Patient characteristics

One hundred twelve JMG patients (42 males, 70 females, male-to-female ratio 0.6) were identified (Additional file 1: Table S1). The average age of onset was 8.5 years (range 0–20 years). The peak age of onset was biphasic: one peak was before the age of 5 and the other peak occurred during adolescence.

The most common symptoms were ptosis in 108 (96.4%) patients followed by diurnal symptom change in 61 (54.5%), muscle weakness in 24 (21.4%), double vision in 23 (20.5%), bulbar palsy in 18 (16.1%), respiratory symptoms in 10 (8.9%), head deviation in 3 (2.7%), ophthalmoplegia in 2 (1.8%), and facial palsy in 1 (0.9%) patient.

Clinical classification of the disease severity at onset was evaluated as class I in 88 (78.6%), class II in 13 (11.64%), class III in 3 (2.7%), class IV in 4 (3.6%) and class V in 4 (3.6%) patients. The age of onset for ocular type MG (*n* = 88) was younger than that for systemic type (*n* = 24) (7.29 ± 6.17 vs. 13.07 ± 4.71, *P* = 0.001). Four patients had a family history of MG. Three patients had a family history of thyroid diseases. Almost all patients were Asian, except one patient was Caucasian.

Serologic tests were performed in 107 (95.5%) patients. Peak anti-acetylcholine receptor antibodies were ≥ 0.5 nmol/L in 60 (56%), 0.2–0.5 nmol/L in 11 (10%), and <  0.2 nmol/L in 36 patients (34%). One seronegative patient had autoantibodies against MuSK and presented with systemic type and bulbar palsy.

Repetitive stimulation tests were performed in 50 patients, and 35 patients exhibited a typical decremental change. No correlation was observed between the anti-acetylcholine receptor value and repetitive stimulation test results.

Two patients had presence of thymoma. The peak serology status of anti-acetylcholine receptor antibodies was 49 and 5.47 each. After excluding them, 53 patients had done chest computed tomography. Thirty-three showed positive thymus hyperplasia, and 22 were negative. No correlation was observed between the anti-acetylcholine receptor value and thymus hyperplasia (Additional file 2: Table S2).

### Outcome at two years and last outpatient clinic follow-up

**Fig. 1:**
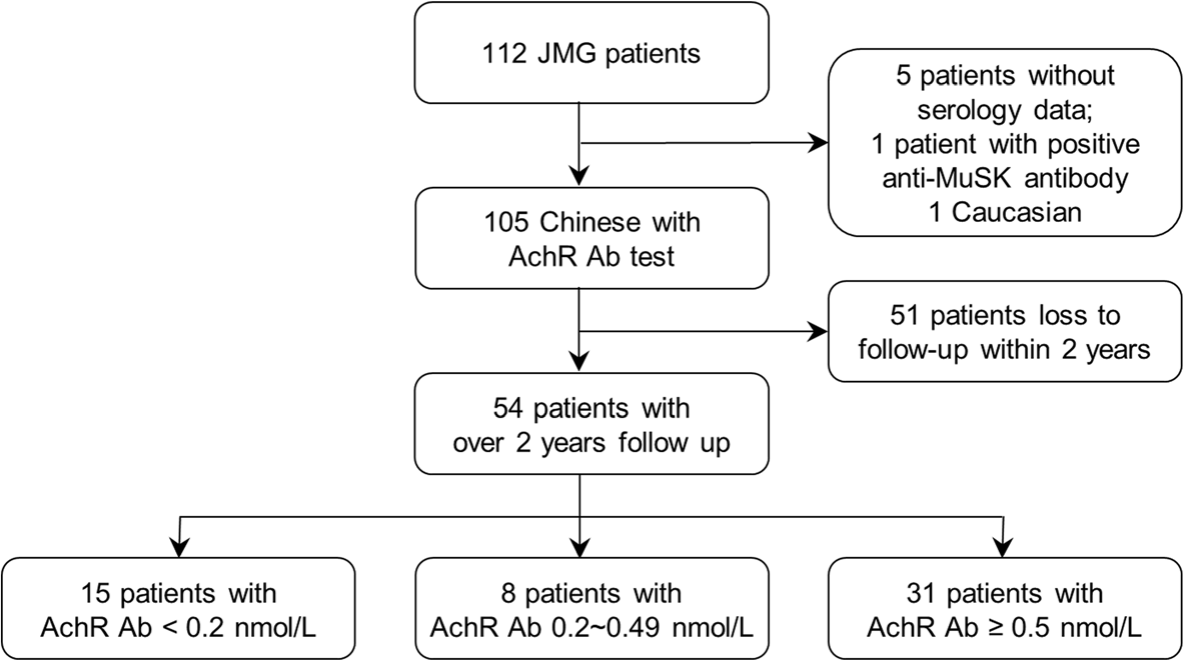
Flowchart of study exclusion criteria. Patients who did not complete follow-up for two years or who had antibodies other than anti-acetylcholine receptor antibodies were excluded

After excluding patients without serum antibody data, one Caucasian, and one patient positive for anti-MuSK antibodies, a total of 54 patients followed up for over 2 years (range 24 to 258 months, mean 112 months) (Fig. 1). We compared the demographic data of JMG patients with different levels of anti-acetylcholine receptor antibodies, and no difference was observed (Table 1). In total, 42 patients (80%) were ocular type and 12 patients (20%) were generalized type. Nine (9/54, 16.7%) achieved complete remission without medication use at 2 years after diagnosis. Thirteen (24.1%) patients achieved complete remission during later follow-up periods. Thirty-two (59.3%) patients had improved symptoms but were maintained on oral medication, 5 (9.3%) patients were maintained on a high dosage of immunosuppressant, and 4 (7.3%) patients had worse symptoms. Fifty-two of 106 (49%) patients had incomplete follow-up over 2 years. No gender, age, disease severity, and intervention differences were observed between patients followed up for 2 years and those without.

**Table 1:** Demographic data of 54 juvenile myasthenia gravis patients with different levels of AchR Ab

		Anti-AchR Ab titer < 0.2 nmol/L (*n* = 15)	Anti-AchR Ab titer ≥0.2 and < 0.5 nmol/L (*n* = 8)	Anti-AchR Ab titer ≥0.5 nmol/L (*n* = 31)	*p*
		n (%)	n (%)	n (%)	
Sex	Female	7 (46)	4 (50)	19 (61)	*0.69*
	Male	8 (54)	4 (50)	12 (39)	
Age	< 10 year	8 (53)	6 (75)	22 (71)	*0.52*
	11~20 year	7 (46)	2 (25)	9 (29)	
Symptoms at onset	Ptosis	15 (100)	8 (100)	29 (94)	*1.00*
	Diurnal change	10 (67)	4 (50)	19 (61)	*0.80*
	Diplopia	5 (33)	2 (25)	4 (13)	*0.44*
	Bulbar sign	3 (23)	0 (0)	5 (19)	*0.73*
	Respiratory symptoms	1 (7)	0 (0)	3 (11)	*1.00*
Clinical subtype	Ocular type	10 (67)	8 (100)	24 (77)	*0.22*
	Generalized type	5 (33)	0 (0)	7 (23)	
	^**a**^MGFA classification				*0.41*
	I	10 (67)	8 (100)	24 (77)	
	II	4 (27)	0 (0)	3 (10)	
	III	1 (6)	0 (0)	1 (3)	
	IV	0 (0)	0 (0)	3 (10)	
	V	0 (0)	0 (0)	0	
Ethnicity	Chinese	15 (100)	8 (100)	31 (100)	*1.00*
Co-morbid diseases	Thymus hyperplasia	6 (40)	3 (37.5)	10 (32)	*0.89*
	Hyperthyroidism	1 (6.7)	1 (13)	6 (19)	*0.66*
	Thymoma	0	0	1 (3)	*NA*
Treatment	Total	14 (93)	8 (100)	31 (100)	*0.42*
	Thymectomy	2 (13)	2 (25)	7 (23)	*0.71*
	Prednisolone only	1 (6.7)	0	2 (6.5)	*1.00*
	Anti-cholinergic regimen only	6 (40)	4 (50)	7 (23)	*0.23*
	Both prednisolone and anti-cholinergic regimen	5 (33)	2 (25)	15 (48)	*0.44*

*Abbreviations*: Anti-AchR Ab, anti-acetylcholine receptor antibody

^**a**^Class I, ocular MG and all other muscle strength is normal; Class II, mild weakness affecting other than ocular muscles; Class III, moderate weakness affecting other than ocular muscles; Class IV, severe weakness affecting other than ocular muscles; Class V, intubation, with or without mechanical ventilation

### Outcome versus anti-acetylcholine receptor antibody levels

**Fig. 2:**
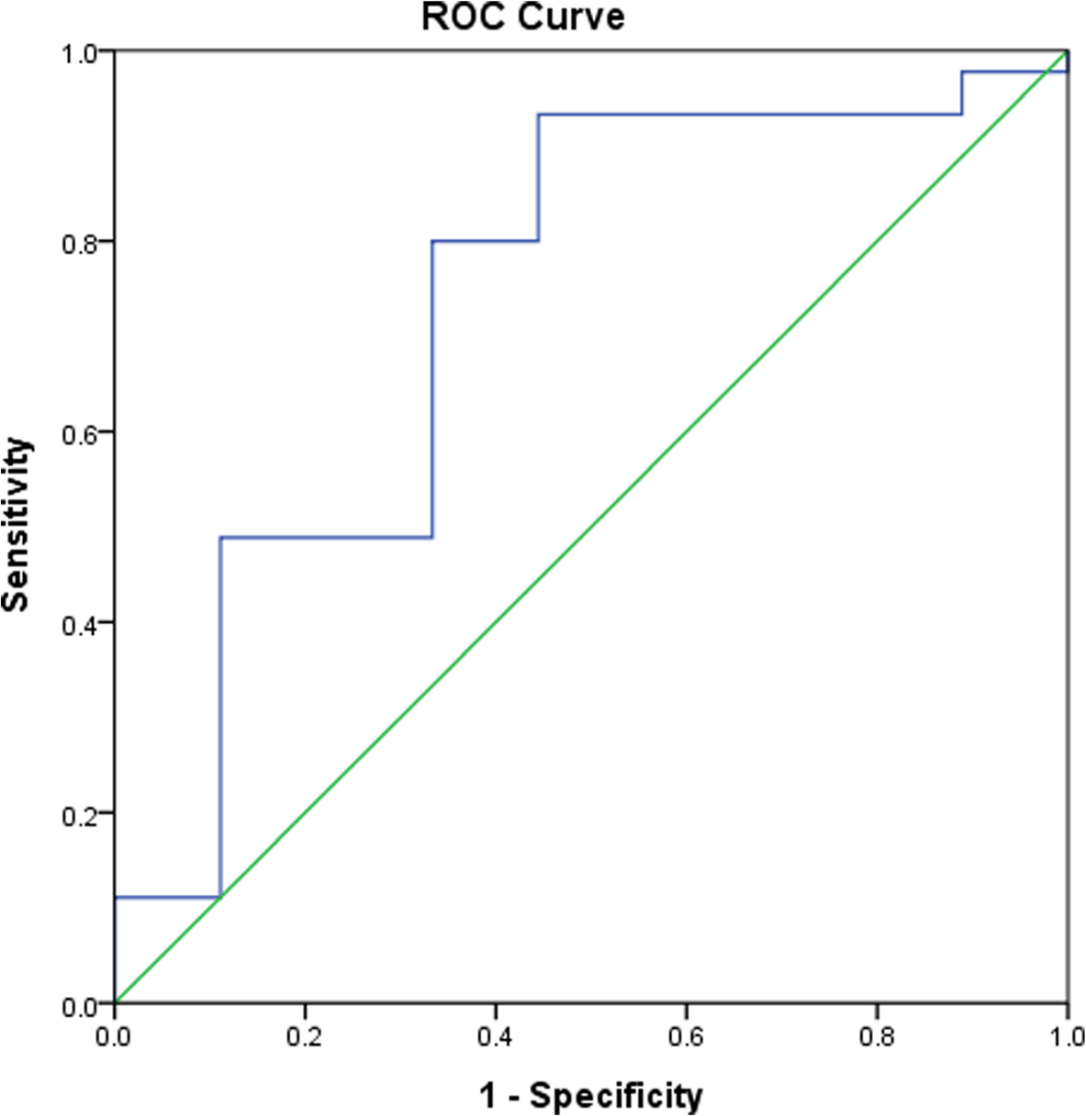
Receiver operating characteristic (ROC) curve of the outcome at 2 years using a cut-off value of 0.2 nmol/L. The area under the ROC curve is 0.73, suggesting fair accuracy

**Fig. 3:**
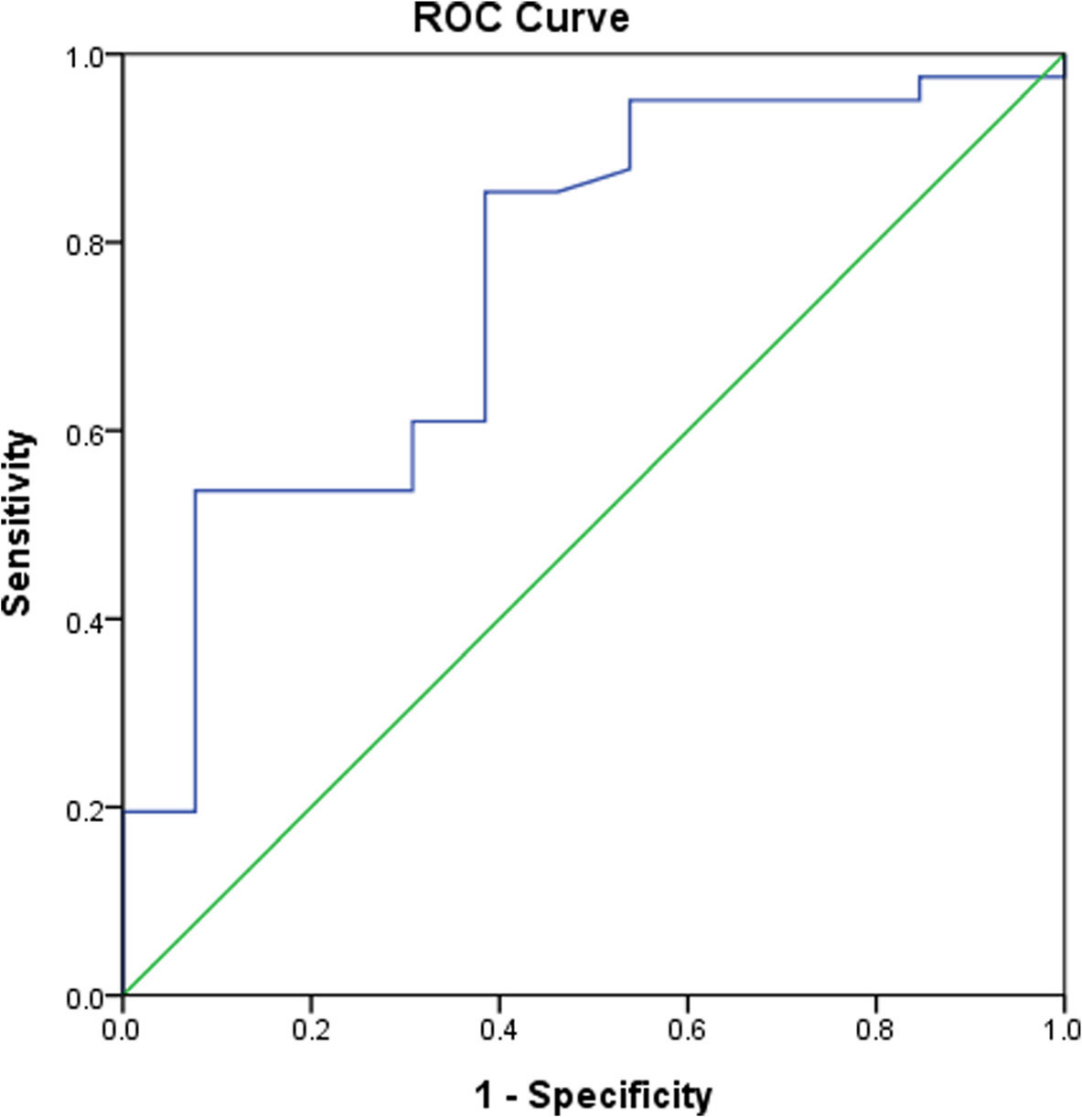
Receiver operating characteristic curve of the outcome at last outpatient clinic using a cut-off value of 0.2 nmol/L. The area under the ROC curve is 0.76, suggesting fair accuracy

**Fig. 4:**
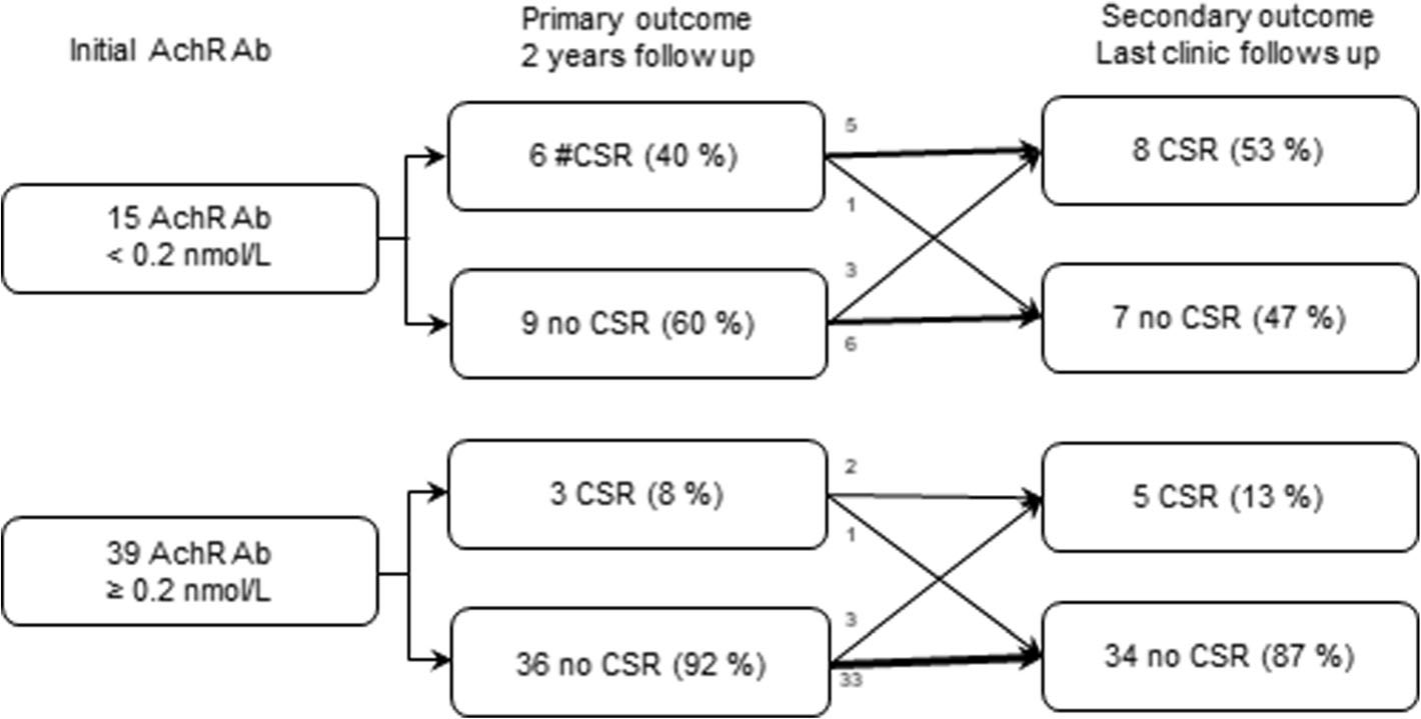
Flowchart of outcomes of juvenile myasthenia gravis evaluated at 2 years and last outpatient clinic (average 9 years) after diagnosis. # complete symptom remission (CSR) rate, the cessation of medication for at least 1 year without symptoms

We compared two-year outcomes among 54 JMG patients using receiver operating characteristic curve (ROC) curves with anti-acetylcholine receptor antibodies at different levels. ROC curves of primary and secondary outcomes are presented in Figs. 2 and 3. Analyses revealed that a serum cut-off level of 0.2 nmol/L serves as an optimal predictive value at 2 years after diagnosis (sensitivity: 80%, specificity: 66%, PPV: 92%, NPV: 40%) (Table 2) and at last outpatient clinic (sensitivity: 83%, specificity: 57%, PPV: 85%, NPV: 53%) (Table 3). JMG patients with negative anti-acetylcholine receptor antibodies (< 0.2 nmol/L) were significantly more likely to achieve complete remission compared with those with antibodies ≥0.2 nmol/L at 2 years (6/15 [40%] vs. 3/39 [7.7%], 95% confidence interval [CI] 1.670 to 38.323) (Table 2, Fig. 4). In addition, JMG patients with anti-acetylcholine receptor antibodies less than 0.2 nmol/L exhibited a higher rate of complete remission compared with those with antibody > 0.2 nmol/L at last out-patient visit (8/15 [53.3%] vs. 5/39 [12.8%], 95% CI 2.367 to 20.704) (Table 3, Fig. 4). After adjustment for age, gender, classification and thymectomy operation, the clinical outcome exhibited a significant difference if we used a serum level of 0.2 nmol/L as a cut-off (Table 4).

**Table 2:** Comparison of AchR Ab cut-off levels associate to outcome at 2 years after diagnosis

	Positive	Negative	*P*	Sen	Spe	PPV	NPV
Use 0.2 as cut-off	n (%)	n (%)		% (confident interval)	% (confident interval)	% (confident interval)	% (confident interval)
No CSR	36 (92)	9 (60)	0.010*	80 (65.4–90.4)	66.7 (29.9–92.5)	92.3 (82.5–96.8)	40 (24.0–58.4)
CSR	3 (8)	6 (40)					
Use 0.5 as cut-off	n (%)	n (%)					
No CSR	28 (93)	17 (71)	*0.148*	62 (46.5–76.2)	66.7 (29.9–92.5)	90.3 (78.3–96.0)	26.1 (16.3–39.0)
CSR	3 (7)	6 (29)					

*Abbreviations*: *AchR Ab* Acetylcholine receptor antibodies, *Sen* Sensitivity, *Spe* Specificity, *PPV* Positive predictive value, *NPV* Negative predictive value, *CSR* Complete symptom remission

**Table 3:** Comparison of AchR Ab cut-off levels associate to outcome at last outpatient after diagnosis

	Positive	Negative	*P*	Sen	Spe	PPV	NPV
Use 0.2 as cut-off	n (%)	n (%)		% (confident interval)	% (confident interval)	% (confident interval)	% (confident interval)
No CSR	34 (87)	7 (47)	0.004*	82.9 (67.9–92.8)	57.1 (28.9–82.3)	85.0 (75.3–91.3)	53.3 (33.6–72.0)
CSR	5 (13)	8 (53)					
Use 0.5 as cut-off	n (%)	n (%)					
No CSR	26 (87)	15 (63)	*0.197*	63.4 (46.9–77.9)	57 (28.9–82.3)	81.2 (69.4–89.2)	34.8 (22.5–49.5)
CSR	5 (13)	8 (37)					

*Abbreviations*: *AchR Ab* Acetylcholine receptor antibodies, *Sen* Sensitivity, *Spe* Specificity, *PPV* Positive predictive value, *NPV* Negative predictive value, *CSR* Complete symptom remission

**Table 4:** Comparison of AchR Ab cut-off levels and outcome using logistic regression

		Unadjusted regression			Adjusted regression		
		OR	95% CI	*P*	OR	95% CI	*p*
No CSR at 2 years after diagnosis	> 0.2 vs. < 0.2	8.0	1.67–38.3	0.009*	7.5	1.26–45.1	0.027*
	> 0.5 vs. < 0.5	3.3	0.72–14.9	*0.122*			
No CSR at last outpatient clinic	> 0.2 vs. < 0.2	7.8	1.95–31.0	0.004*	6.3	1.42–27.5	0.015*
	> 0.5 vs. < 0.5	2.8	0.77–10.0	*0.120*			

*Abbreviations*: *AchR Ab* Acetylcholine receptor antibodies, adjusted of sex, age, class and thymectomy operation

### Clinical fluctuations

Four boys (aged 0.5 years, 1.5 years, 2.5 years and 7.3 years) with initial anti-acetylcholine levels less than 0.2 nmol/L became positive later (0.25 nmol/L, 12.5 nmol/L, 2.19 nmol/L and 11.1 nmol/L). The occurrence of seroconversion was detected 11, 19, 107, and 85 months after clinical onset, respectively. One of the 4 patients achieved complete remission at 2 years but developed symptom relapse, and the other 3 were maintained on oral medication throughout the study course.

### Comorbidity

Comorbid diseases included Graves’ disease in 11 (9.8%) patients, Hashimoto disease in 2 (1.8%), thymoma in 2 (1.8%), systemic lupus erythematous in 1 (0.9%), and rheumatic arthritis in 1 (0.9%) patient.

We analyzed the characteristics of 13 patients with comorbid autoimmune thyroid disease. Nine (69%) patients were females, and the mean onset age was older than JMG patients without comorbid autoimmune thyroid disease (11.8 ± 5.8 years old vs. 8.0 ± 6.3 years old, 95% CI 0.018 to 7.33). Ten (77%) patients had ocular type MG. Two had a family history of hyperthyroidism, and two had a family history of MG. In 10 (77%) patients, comorbid autoimmune thyroid diseases were diagnosed at or after the JMG diagnosis was made. Patients with anti-acetylcholine antibody serum levels < 0.2 nmol/L were less likely to have autoimmune thyroid disease (1/35 [2.9%] vs. 12/71 [16.9%], 95% CI 0.018 to 1.161). No correlation found using a cut-off level of 0.5 nmol/L.

### Pharmacologic and surgical treatment

Forty-six of 112 (41.1%) patients received both cholinesterase inhibitors and steroids, 35 (31.3%) patients received cholinesterase inhibitors only, 5 (4.5%) patients received steroids only, and 8 (7.1%) patients did not receive any medication.

Eighteen of 112 (11%) patients received thymectomy, and 16 (16/18, 90%) were female. Patients manifesting with initial systemic MG were more likely to require thymectomy than those with initial ocular MG (12/24 [50%] vs. 6/88 [6.8%], 95% CI 4.31 to 43.49). No difference in the primary and secondary outcomes was observed between patients who received both or either cholinesterase inhibitors and steroids or who underwent thymectomy.

## Discussion

Our study demonstrated that overall prognosis for remission is fair at 2 years since diagnosis (9/54, 16.7%) and in a longer clinical observation (13/54, 23.6%) in JMG. The remission rate in our cohort is compatible to previous studies, demonstrating that the remission rate in JMG was approximately 22.2 to 30% depending on the follow-up period [1, 2, 6, 7, 12]. We found that JMG patients with anti-acetylcholine receptor antibodies less than 0.2 nmol/L exhibited increased complete remission rate both at 2 years and even during a longer observation period. Furthermore, the optimal predictive value for clinical outcomes was found using cut-off value of 0.2 nmol/L but not 0.5 nmol/L. To the best of our knowledge, the current study is the first to apply the absence of anti-acetylcholine receptor antibodies as a predictive parameter for JMG patients.

Measurement of anti-acetylcholine receptor antibodies is important to establish the diagnosis. The relationship between antibodies level and outcome has been discussed. Afifi and Bell et al. [9]. first studied 17 JMG patients and revealed that the negative serology group likely became medication-free (2/6 [33%] vs. 2/11 [18%], *P* = 0.58), but the result was not significant. A larger study group from Andrew et al. revealed no difference in frequency of remission between anti-acetylcholine receptor antibody-positive and antibody-negative patients [1]. Nevertheless, the study of Andrew’s overall remission rate was relatively lower, and our study participants were Chinese patients. The ethnic origin of the patients influences the acetylcholine receptor antibodies serum level and clinical outcome. Another adult MG study revealed that female sex, late onset and positivity for anti-acetylcholine receptor antibodies were associated with progression from ocular MG to generalized MG [14]. Some of seronegative patients had other antibodies, such as cortactin and lipoprotein receptor-related protein 4. Result revealed these patients have better outcome [5]. This might explain potentially better outcome of our seronegative JMG.

Different cut-off values of anti-acetylcholine receptor antibodies were used in our study to predict outcome. We demonstrated that 0.2 had a better predictive value compared with 0.5. Although the cut-off value of 0.5 nmol/L is now widely used, as 0.2 nmol/L was used previously, for diagnosis of the MG population due to high specificity up to 99.99% [15], nonwhite MG sera were excluded in the study for analysis of the cut-off value. In addition, the age of included patients indicates that most patients had adult onset MG. Chiu et al. demonstrated that Chinese patients with MG had lower anti-acetylcholine receptor antibody values, accounting for the increased frequency of ocular cases compared with that noted in Caucasians [12]. Another recent study enrolled 327 JMG Chinese patients and found the anti-acetylcholine receptor antibody level was reduced in ocular type MG compared with the generalized type [10]. However, these studies did not identify a clear correlation between antibody level and prognosis. We proposed 0.2 nmol/L as a better predictive cut-off value for JMG in the Chinese population.

Factors related to remission have been discussed in many studies. Prepubertal children exhibit increased rates of spontaneous remission compared with postpubertal individuals [1]. Patients who received thymectomy also likely exhibit a better prognosis [6, 12]. MuSK myasthenia gravis also had an increased risk of respiratory crises [4]. We excluded one patient positive for anti-MuSK antibodies from multiple logistic regression to adjust for factors, including age, sex, thymectomy and disease severity, and the result remained significant. Anti-acetylcholine receptor antibodies levels were related to the remission rate in 2 years and longer follow-up.

Four patients had initial serology less than 0.2 nmol/L and then underwent seroconversion with recurrent symptoms or persist medication use. A previous study demonstrated that spontaneous elevation or normalization of antibodies was not uncommon. Up to 41% of the seronegative patients may become seropositive in 1 to 5 years as reported by Anlar et al. [11]. We used the peak serology for the primary exposure variable to avoid “false negative”. The re-evaluation of serum antibodies against the acetylcholine receptor might be helpful if patients had initial anti-acetylcholine receptor antibody values less than 0.2 nmol/L but had persist symptoms or recurrent symptoms.

The most common autoimmune disease comorbid with JMG in our study was autoimmune thyroid disease, which was similar to previous study [6, 16–18]. We observed that the characteristic features of JMG comorbid with autoimmune thyroid disease were female patients, older onset of symptoms, and positive family history of autoimmune disease. We also observed that anti-acetylcholine receptor antibodies less than 0.2 nmol/L were less likely to be comorbid with autoimmune thyroid disease. In contrast, Chen et al. demonstrated that adult MG patients with autoimmune thyroid disease exhibited a slightly lower seropositive rate for anti-acetylcholine receptor antibody (70.2% vs. 75.5%) compared with patients without autoimmune thyroid disease [18]. This finding indicates that the pathogenesis of MG might be different between juvenile and adult onset.

This study has some limitations. First, some antibodies were measured post-immune therapy. In addition, the immunotherapy factor was not adjusted given the small sample size. Second, immunoassays for other antibodies, including muscle-specific kinase, cortactin and lipoprotein receptor-related protein 4, were not routinely performed in our hospital. In addition, Cruz et al. used cell-based assays to demonstrate that serum negative patients may exhibit an improved diagnosis of clustered anti-acetylcholine receptor antibodies in serum negative MG [19]. Third, 4 of 15 JMG patients were negative for serum antibodies but eventually yield an equivocal or positive result. We require an even longer observation period and a larger population to estimate the true negative group in JMG. Fourth, lack of facility of genetic test for congenital myasthenia syndrome in our hospital.

## Conclusions

Our findings demonstrated that anti-acetylcholine receptor antibodies are not only a diagnostic marker but also an acceptable predictive marker for outcome in patients with JMG. We suggest that patients with peak anti-acetylcholine receptor antibody levels less than 0.2 nmol/L exhibited better prognosis and less comorbid autoimmune thyroid diseases among Chinese.

## Additional files

Additional file 1: **Table S1.** Clinical features of generalized and ocular JMG (DOCX 39 kb)

Additional file 2: **Table S2.** Clinical Features of chest computed tomography of JMG (DOCX 25 kb)

## Data Availability

The datasets used and analysed during the current study are available from the corresponding author on reasonable request.

## Abbreviations

CSR: Complete symptom remission
JMG: Juvenile myasthenia gravis
MG: Myasthenia gravis
MuSK: Muscle-specific kinase
NPV: Negative predictive values
PPV: Positive predictive values
ROC: Receiver operating characteristic
