# Supplementary material for: Correlation of anti-acetylcholine receptor antibody levels and long-term outcomes of juvenile myasthenia gravis in Taiwan: a case control study
Source: BMC Neurol. 2019 Jul 18;19:170. doi: 10.1186/s12883-019-1397-0 (PMC6637626; doi:10.1186/s12883-019-1397-0)
Supplement: Supplementary file 1 — Table S1. Clinical features of generalized and ocular JMG (DOCX 39 kb) [file 12883_2019_1397_MOESM1_ESM.docx]

| **Supplementary Table 1. Clinical features of generalized and ocular JMG** | | | | | |
| --- | --- | --- | --- | --- | --- |
|  |  | Generalize JMG | Ocular JMG | *p* |  |
|  |  | n = 24 (%) | n = 88 (%) |  |  |
| **Sex** | Female | 18 (75) | 52 (59) | *0.23* |  |
|  | Male | 6 (25) | 36 (41) |  |  |
| **Age** | <10 year | 6 (25) | 63 (72) | *< 0.001*  * |  |
|  | 11~20 year | 18 (75) | 25 (28) |  |  |
| **Symptoms at onset** | Ptosis | 21 (88) | 87 (99) | *0.03*  * |  |
|  | Diurnal change | 12 (50) | 49 (57) | *0.65* |  |
|  | Diplopia | 8 (33) | 15 (17) | *0.15*  * |  |
|  | Bulbar sign | 15 (62) | 3 (3) | *< 0.001* |  |
|  | Respiratory symptoms | 10 (42) | 0 (0) | *< 0.001*  * |  |
|  | Head deviation | 0 (0) | 3 (3) | *1.00* |  |
|  | Ophthalmoplegia | 1 (4) | 2 (2) | *1.00* |  |
|  | Photophobia | 1 (4) | 0 (0) | *1.00* |  |
|  | Facial palsy | 0 (0) | 1 (1) | *1.00* |  |
| **MGFA^#^ Classification** | I | 0 (0) | 88 (100) |  |  |
|  | II | 13 (54) | 0 (0) |  |  |
|  | III | 3 (12) | 0 (0) |  |  |
|  | IV | 4 (12) | 0 (0) |  |  |
|  | V | 4 (12) | 0 (0) |  |  |
| **Family history** | Myasthenia gravis | 0 (0) | 4 (5) | *1.00* |  |
|  | Thyroid disease | 1 (4) | 2 (2) | *1.00* |  |
| **Ethnicity** | Caucasian | 0 (0) | 1 (1) | *0.52* |  |
|  | Chinese | 24 (100) | 87 (99) |  |  |
| **Co-morbid diseases** | Hyperthyroidism | 2 (8) | 9 (10) | *1.00* |  |
|  | Hypothyroidism | 1 (4) | 1 (1) | *1.00* |  |
|  | Systemic lupus erythema | 1 (3) | 0 (0) | *1.00* |  |
|  | Rheumatic arthritis | 0 (0) | 1 (1) | *1.00* |  |
| **Examination** |  |  |  |  |  |
| Repetitive stimulation test | Positive | 15 (79) | 20 (65) | *0.35* |  |
|  | Negative | 4 (21) | 11 (35) |  |  |
| Ice packing test | Positive | 0 (0) | 3 (43) | *1.00* |  |
|  | Negative | 1 (100) | 4 (57) |  |  |
| Anti-AchR Ab | ≥ 0.5 nmol/L | 14 (61) | 46 (55) | *0.73* |  |
|  | ≥ 0.2 and < 0.5 nmol/L | 1 (4) | 10 (12) |  |  |
|  | < 0.2 nmol/L | 8 (35) | 28 (33) |  |  |
| Anti-MuSK Ab | Positive | 1 (4) | 0 (0) | *1.00* |  |
| Tensilon test | Positive | 3 (75) | 4 (50) | *0.58* |  |
|  | Negative | 1 (25) | 4 (50) |  |  |
| Chest CT | Thymus hyperplasia | 14 (70) | 22 (63) | *0.67* |  |
|  | No thymus hyperplasia | 6 (30) | 13 (37) | * |  |
| **Treatment** | Total | 24 (100) | 80 (91) | *< 0.001* |  |
|  | Thymectomy | 12 (50) | 6 (7) | *< 0.001*  * |  |
|  | Prednisolone only | 0 (0) | 5 (6) | *0.58* |  |
|  | Anti-cholinergic regimen only | 3 (12) | 32 (36) | *0.03* |  |
|  | Both prednisolone and anti-cholinergic regimen | 9 (38) | 37 (42) | *0.82* |  |
| **Thymus pathology** | Total | 12 (50) | 6 (7) | *0.28* |  |
|  | Lymphoid hyperplasia | 7 (58) | 3 (50) | *1.00* |  |
|  | Thymoma | 2 (17) | 0 (0) | *0.53* |  |
|  | Non-specific | 3 (25) | 3 (50) | *0.34* |  |
| **Outcome** |  |  |  |  |  |
| 2 year after diagnosis | CSR | 2 (15) | 7 (16) | *1.00* |  |
|  | No CSR | 11 (85) | 35 (84) |  |  |
| Last clinic follows up | Total | 13 (43) | 42 (48) | *0.55* |  |
|  | CSR | 4 (31) | 9 (21) | *0.48* |  |
|  | Improved symptoms | 8 (62) | 25 (60) | *1.00* |  |
|  | Unchanged/Worse | 1 (7) | 8 (19) | *0.67* |  |
| **Abbreviations: JMG, juvenile myasthenia gravis; Anti-AchR Ab, anti-acetylcholine receptor antibody; Anti-MuSK Ab, anti-muscle-specific tyrosine kinase antibody; CT, computed tomography; CSR, complete symptom remission**  **p < 0.05*  **^#^**Class I, ocular MG and all other muscle strength is normal; Class II, mild weakness affecting other than ocular muscles; Class III, moderate weakness affecting other than ocular muscles; Class IV, severe weakness affecting other than ocular muscles; Class V, intubation, with or without mechanical ventilation. | | | | | |

*
