# Supplementary material for: Correlation of anti-acetylcholine receptor antibody levels and long-term outcomes of juvenile myasthenia gravis in Taiwan: a case control study
Source: BMC Neurol. 2019 Jul 18;19:170. doi: 10.1186/s12883-019-1397-0 (PMC6637626; doi:10.1186/s12883-019-1397-0)
Supplement: Supplementary file 2 — Table S2. Clinical Features of chest computed tomography of JMG (DOCX 25 kb) [file 12883_2019_1397_MOESM2_ESM.docx]

| **Supplementary Table 2. Clinical Features of chest computed tomography of JMG** | | | | | |
| --- | --- | --- | --- | --- | --- |
|  |  | Thymus hyperplasia | No thymus hyperplasia |  |  |
|  |  | n = 33 (%) | n = 20 (%) | *p* |  |
| **Sex** | Female | 19 (58) | 16 (80) | *0.14* |  |
|  | Male | 14 (42) | 4 (20) |  |  |
| **Age** | <10 year | 17 (52) | 10 (50) | *1.00* |  |
|  | 11~20 year | 16 (48) | 10 (50) |  |  |
| **MGFA^#^ Classification** | I | 21 (64) | 14 (70) | *0.76* |  |
|  | II | 7 (21) | 4 (20) |  |  |
|  | III | 3 (9) | 0 (0) |  |  |
|  | IV | 1 (3) | 1 (5) |  |  |
|  | V | 1 (3) | 1 (5) |  |  |
| **Examination** |  |  |  |  |  |
| Repetitive stimulation test | Positive | 17 (81) | 7 (64) | *0.40* |  |
|  | Negative | 4 (19) | 4 (36) |  |  |
| Anti-AchR Ab | ≥ 0.5 nmol/L | 15 (47) | 14 (70) | *0.23* |  |
|  | ≥ 0.2 and < 0.5 nmol/L | 5 (16) | 1 (5) |  |  |
|  | < 0.2 nmol/L | 12 (37) | 5 (25) |  |  |
| Anti-MuSK Ab | Positive | 1 (3) | 0 (0) | *1.00* |  |
| **Pathology** | Lymphoid hyperplasia | 10 (77) | 0 (0) | *0.10* |  |
|  | Non-specific | 3 (23) | 2 (100) |  |  |
| **Treatment** | Total treatment | 32 (97) | 20 (100) | *0.11* |  |
|  | Thymectomy | 13 (40) | 2 (10) | *0.028*  * |  |
|  | Prednisolone only | 1 (3) | 1 (5) | *1.00* |  |
|  | Anti-cholinergic regimen only | 8 (24) | 7 (35) | *0.53* |  |
|  | Both prednisolone and anti-cholinergic regimen | 10 (30) | 10 (50) | *0.24* |  |
| **Outcome** |  |  |  |  |  |
| 2 year after diagnosis | CSR | 1 (5) | 1 (9) | *1.00* |  |
|  | No CSR | 18 (95) | 10 (91) |  |  |
| Last clinic follows up | Total | 19 (58) | 11 (55) | *0.12* |  |
|  | CSR | 5 (26) | 2 (18) | *1.00* |  |
|  | Improved symptoms | 13 (69) | 5 (46) | *0.27* |  |
|  | Unchanged/Worse | 1 (5) | 4 (36) | *0.05* |  |
| **Abbreviations: Anti-AchR Ab, anti-acetylcholine receptor antibody; Anti-MuSK Ab, anti-muscle-specific tyrosine kinase antibody; CSR, complete symptom remission**  **p < 0.05*  **^#^**Class I, ocular MG and all other muscle strength is normal; Class II, mild weakness affecting other than ocular muscles; Class III, moderate weakness affecting other than ocular muscles; Class IV, severe weakness affecting other than ocular muscles; Class V, intubation, with or without mechanical ventilation. | | | | | |
